# Health care delivery model in India with relevance to diabetes care

**DOI:** 10.1016/j.heliyon.2022.e10904

**Published:** 2022-10-04

**Authors:** Ashok Kumar Das, Banshi Saboo, Anuj Maheshwari, Mohanan Nair V, Samar Banerjee, Jayakumar C, Benny P. V, Sunil Prasobh P, Anjana Ranjit Mohan, Vasudevan Sambu Potty, Jothydev Kesavadev

**Affiliations:** a Department of Endocrinology, Pondicherry Institute of Medical Sciences, Pondicherry, Tamil Nadu, India; b Diacare, Diabetes Care & Hormone Clinic, Ahmedabad, India; c Shri Hari Kamal Diabetes & Heart Clinic, Lucknow, India; d Kandala Cooperative Hospital, Trivandrum, Kerala, India; e Dept. of Medicine, Vivekananda Institute of Medical Sciences, Kolkata, West Bengal, India; f Department of General Medicine, Sree Gokulam Medical College and Research Foundation, Trivandrum, Kerala, India; g Department of Community Medicine, Sree Gokulam Medical College and Research Foundation, Trivandrum, Kerala, India; h Department of Internal Medicine, Government Medical College, Kollam, Kerala, India; i Department of Diabetology, Dr. Mohan's Diabetes Specialities Centre, Chennai, India; j Department of Urology, Government Medical College, Trivandrum, Kerala, India; k Department of Diabetology, Jothydev's Diabetes Research Centers, Trivandrum, Kerala, India

**Keywords:** Diabetes, Health care model, Patient adherence, India, Health care policies

## Abstract

The Indian healthcare scenario presents a spectrum of contrasting landscapes. Socioeconomic factors, problems with medical infrastructure, insufficiency in the supply of medical requisites, economic disparities due to major differences in diabetes care delivery in the government and private sectors and difficulty in accessing quality health care facilities challenges effective diabetes care in India. The article gives insights into the practical solutions and the proposed White paper model to resolve major challenges faced by the Indian diabetes care sector for effective diabetes care delivered at Jothydev's Diabetes Educational Forum Global Diabetes Convention 2019.

## Highlights

• The prevalence of diabetes in India is on its rise.
• Socioeconomic factors, lack of diagnosis etc challenges Indian diabetes care system.
• Health care models appropriate for Indian scenario should be developed.
• Public-Private partnership is required for effective health care delivery.

## 1 Introduction

Diabetes is a health-related global concern that threatens the economy of developing nations, especially India. India's large population and socio-economic transitions have made it an epicenter of diabetes. In 2017, diabetes alone exhausted about 5–25% share of an average Indian household earnings by spending about 31 billion US dollars on diabetes management [1]. Several challenges contribute to diabetes among Indians, including unawareness about diabetes and its complications, inadequate healthcare facilities and lack of access, healthcare expenditure, non-adherence to treatment, and lack of accountability.

Collective and active involvement of healthcare professionals (HCPs) and stakeholders involved in diabetes care is required to build a focused multidisciplinary approach. Some of the government and/or private organizations have tried to implement effective educational programs for increasing awareness about the proper management of diabetes [2]. Despite these promising approaches, attention and improvisation of efforts are essential for developing a healthcare system that delivers quality diabetes care. Delivering accessible, affordable, and high-quality care are some of the major challenges it faces and therefore healthcare delivery systems must identify more proactive ways to manage patients in the community and in their homes.

Jothydev's Professional Education Forum (JPEF) Annual Diabetes Convention is an international diabetes convention with the global participation of diabetes care professionals from all streams. JPEF Global Convention 2019 organized a discussion forum including diabetes experts from India who are actively involved in patient care either in clinical practice or in diabetes administration. The experts proposed a “White paper model” to sort out the challenges faced during diabetes management in India.

The article aims to emphasize how various aspects of diabetes care delivery can be congregated to develop a “White paper model” which will harmonize the development of the most appropriate health care delivery model for India.

## 2 Diabetes burden in India

According to the International Diabetes Federation (IDF), about 77 million of Indians aged 20–79 years are currently living with diabetes, and the number is expected to increase to 134.2 million by 2045. It is estimated that more than 1 million deaths are attributed to diabetes and diabetes-related complications in India alone. These huge numbers rank India as the first in the world with the highest socio-economic diabetes burden. It is estimated that in low-income families, the cost of diabetes treatment for an adult with diabetes is up to 25 per cent of the family income. A study conducted by Anjana et al in 15 states of India reported that the mean prevalence of prediabetes in India is 10.3% (state-wise range, 6.0–14.7%) [3, 4] Survey studies revealed that about 10–16% of the urban and 5–8% of the rural populations have diabetes [5, 6].

According to the data published by IDF in 2019, 43.9 million people with diabetes are undiagnosed in India because of the poorly developed healthcare systems (https://www.diabetesatlas.org/upload/resources/material/20200302_133351_IDFATLAS9e-final-web.pdf). The number is quite alarming from an Indian perspective as the progression from pre-diabetes to diabetes occurs much faster in the Asian population than in any other ethnic population [7, 8]. The worrisome fact is that the prevalence is increasing in children and young adults.

## 3 Poor outcome and type 2 diabetes mellitus (T2DM) complications as an indicator of insufficient care and monitoring

Mounting evidence from studies conducted in India highlighted the fact that the percentage of people with diabetes with poor glycemic control (HbA1c >8%), uncontrolled hypertension, dyslipidemia, and vascular complications related to diabetes is greater than 50% [9, 10]. that vary across regions. The ICMR-INDIAB study had shown that the prevalence of undiagnosed hypertension, and dyslipidemia is high and the level of glycemic control among subjects with self-reported diabetes in India is poor [11, 12, 13]. A study from Maharashtra showed that even with the use of pharmacological agents HbA1c was found to be >7% in 72.7% of patients tested. being on pharmacological agents. Equivalently, studies conducted among PWD from Madhya Pradesh (n = 540) reported that 72% still had an HbA1c ≥ 7% while on oral antidiabetic agents (OAD), on insulin and both OAD and insulin [14]. The Delhi Diabetes Community (DEDICOM) survey including adults with diabetes (n = 819) from middle and upper socioeconomic backgrounds showed a very low percentage of PWD (13%) had monitored their previous year's HbA1c. The mean frequency of self-monitoring of blood glucose (SMBG) was estimated as 3.1/month with approximately >40% with HbA1c> 8% [9]. Further studies revealed that poor diabetes management leads to various micro and macrovascular complications, which largely contributes to the increased risk of mortality and morbidity rates among PWD. Mohan et al in their study reported that the prevalence of Coronary Artery Disease (CAD) was 21.4% among PWD compared to 9.1% in subjects with normal glucose tolerance in the scenario where the chances of occurrence of CAD even before the onset of diabetes' [15].

## 4 Challenges in health care delivery for diabetes in India

In India, diabetes management becomes arduous due to various reasons such as diabetes unawareness among the community, poor access to quality care, scarcity of trained staff, monitoring facility, and the unavailability of antidiabetic drugs.

### 4.1 Low awareness and diagnosis of diabetes

Awareness of diabetes and its complications plays a vital role in successful disease management. Several studies analyzed the relationship between diabetes awareness and undiagnosed diabetes levels among the Indian population. The ICMR-INDIAB (Phase I) reported that only 43.2% of the population were aware of diabetes, and the level of awareness was significantly low among rural residents compared to urban residents (36.8% vs. 58.4%, p < 0.001) [16]. A study from Western India (n = 400) reported that 9.4% of the population had good awareness, 71.3% had moderate and 18.2% patients had low awareness of diabetes [17]. Another study from South India revealed good awareness and positive attitude in 55.6% and 52.8% people respectively. Among 25.4% of the people known to have diabetes, only 40.7% had good knowledge, 53.8% had a positive attitude, and 57.6% had good practice patterns [18]. A study by Deepa et al which assessed the awareness rate of complications among PWD, reported that 51.5% of the general population and 72.7% of population with diabetes knew that diabetes could affect other organs [19]. Also, low socio-economic position (SEP) measured by educational level, occupation and income increases the risk of diabetes especially T2D [20, 21] These studies highlight the importance of enhancing the awareness level about diabetes, associated comorbidities, and management among PWD and the general population.

### 4.2 Lack of diagnosis and access to quality care

In India, the number of undiagnosed cases is high and the diagnosis is often made incidentally through abnormal urine or blood glucose tests or from other associated complications. It is estimated that >50% of diabetes cases in rural areas, and about 30% in urban areas remain undiagnosed. A cross-sectional house-to-house survey from the Gwalior-Chambal region of India involving 7608 subjects aged 20–79 years reported that about 50% of the PWD among the rural population were diagnosed for the first time [22]. In India, the unknown-to- known diabetes ratio is higher (3.3:1) for rural areas compared to urban areas (about 1.8:1) [23]. Treatment expenditure burden, logistic issues for patients living in rural settings, underdeveloped public health-care infrastructure, and unavailability of antidiabetic medications may contribute to the higher rate of unknown-to-known diabetes ratio and mortality rate in rural areas [24]. Despite including insulin in the National List of Essential Medicines, the most common cause of death in type 1 diabetes in India is lack of access to insulin [25]. The uncertainty in the availability of medicines and the lack of price control over the private sector, also adds to the poor diabetes treatment outcomes. For the prevention and control of major non communicable diseases (NCDs), the National Programme for Prevention and Control of Cancer, Diabetes, Cardiovascular Diseases and Stroke (NPCDCS) was launched in 2010 with focus on strengthening infrastructure, human resource development, health promotion, early diagnosis, management and referral. Under NPCDCS, NCD Cells are being established at National, State and District levels for programme management, and NCD Clinics are being set up at District and CHC levels, to provide services for early diagnosis, treatment and follow-up for common NCDs. Provision has been made under the programme to provide free diagnostic facilities and drugs for patients attending the NCD clinics (https://www.nhm.gov.in/index1.php?lang=1&amp;level=2&amp;sublinkid=1048&amp;lid=604). Similar to NPCDCS is the National Diabetes Control Programme initiated by the Government of India on pilot basis during the seventh five-year plan in 1987 in some districts of Tamil Nadu, Jammu & Kashmir and Karnataka, but due to paucity of funds in subsequent years this programme could not be expanded further in remaining states (http://pib.nic.in/release/release.asp?relid=34389). The National Programme for Health Care of the Elderly (NPHCE) is an articulation of the International and national commitments of the Government as envisaged under the UN Convention on the Rights of Persons with Disabilities (UNCRPD), National Policy on Older Persons (NPOP) adopted by the Government of India in 1999 and has envisaged providing promotional, preventive, curative and rehabilitative services in an integrated manner for the elderly (nhp.gov.in).

### 4.3 Medication adherence and patient issues

Poor medication adherence is a well-documented challenge in patients with diabetes. Poor adherence not only compromise clinical outcomes in PWD, but also increases healthcare costs, complications, hospitalizations, and mortality rates. Specific factors identified as key contributors to poor medication adherence were medication costs, regimen complexity, lack of transportation, long wait times at the pharmacy, patient's emotional well-being, and patient beliefs and fears (due to the fact of fear of insulin and dependence on medications or insulin etc.) [26, 27]. Basu et al in their study conducted in a tertiary care government hospital in Central Delhi (n = 375) reported non-compliance to medications in 17.6% of PWD, and suboptimal glycemic control in 69% of patients (n = 259) on insulin therapy. The authors also reported missed clinic appointments and suboptimal refill adherence in 71.2% of patients due to out of pocket expenditure on medications and travel [28]. Studies from a rural hospital of Western India (n = 307) and Southern India (n = 162) had reported 23.7% and 95.6% compliance rate respectively in patients with diabetes [17, 29]. Indeed, the poor clinical outcomes experienced by patients with diabetes may be due, at least in part, to poor medication adherence.

### 4.4 Time constraints and suboptimal knowledge among physicians

Suboptimal knowledge of or improper adherence to the recommended guidelines, constraints of time and facilities, and attitude are the major issues observed in India, which lead to improper management of blood glucose levels and other complications. However, a survey of clinical diabetologists showed that low awareness among physicians (22.7%), non-applicability of Western guidelines in Indian patients (22.7%), and treatment cost (18.2%) are the existing barriers to the practice of evidence-based diabetes management. Poor referrals to endocrinologists, and other specialists, lack of counseling, and lack of practice of evidence-based medicine impede the efficiency of patient care in India. These factors may also lead to poor patient compliance which in turn compromises safety and treatment effectiveness and hence may contribute to increase in morbidity and mortality rates. Also, general practitioners (GPs) getting certified as diabetologists from low-level universities and colleges and lack of requirement of national qualification for practice affect the regulation of quality diabetes care.

### 4.5 Lack of trained diabetes educators and disparity in funding

According to the American Diabetes Association (ADA) recommendations, measurement and monitoring of the key outcomes of Diabetes Self-Management Education and Support (DMSE/S) including self-management, clinical outcomes, health status, and quality of life are essentially required for appropriate diabetes management [30]. The lack of trained diabetes educators in India not only increases the burden on physicians to educate patients but also leads to an increase in the incidence and prevalence of T2DM. It is also documented that dietitians in India were generally unfamiliar with the details of the process used to identify, evaluate, and synthesize the research into the guidelines [31].

In India, the wide disparity in funding for diabetes and healthcare facilities exist due to variable levels of awareness of diabetes and the economic impact of diabetes care on the people and government.

## 5 Suggested measures to improve health care delivery in India

A concerted and multidisciplinary approach in treating diabetes has been recognized as a beneficial strategy to prevent the rising prevalence of diabetes-associated complications in India. Government initiatives are essentially required to deliver quality care to the maximum number of PWD. Various health care models, focusing on reorganizing the health care services with better-designed delivery systems, have been tested to improve clinical outcomes in patients with diabetes [32]. Specific interventions for provider education, patient education, financial incentives, feedback, and reminders have also been found useful in some studies [33].

### 5.1 Education and training

Primary care is the first point of contact between patients and health system which delivers whole-person care for health needs. In India, lack of trained and experienced physicians at primary level health facilities is a major limitation. Trained healthcare staff is a prerequisite to deliver quality care and establish a referral linkage system with the secondary/tertiary level [34]. Ethiopia has tested one of the models for health delivery to patients with chronic illness, which provides specialized support of once a month to nurse-led primary health centers [35]. The patient-centered approach reduces long-term diabetes complications with the timely involvement of trained diabetes educators, community involvement, and maintenance of treatment registers. The Indian National Programme for Prevention and Control of Cancer, Diabetes, Cardiovascular Diseases and Stroke (NPCDCS) recommends training of nurses as diabetes educators. The ten principles of care for people with diabetes or at risk for diabetes framed by the National Diabetes Education Program (NDEP) simplify the approach to diabetes. The first seven of the guiding principles highlight the steps to be implemented in the primary care physician level to alleviate the diabetes burden. The ten guiding principles of NDEP (https://www.aafp.org/news/health-of-the-public/20180905ndepprinciples.html) are listed as follows:

Principle 1: Identify People with Undiagnosed Diabetes and Prediabetes
Principle 2: Manage Prediabetes to Prevent or Delay the Onset of Type 2 Diabetes
Principle 3: Provide Comprehensive, Patient-centered Diabetes Care
Principle 4: Provide Ongoing Self-management Education and Support for People with Diabetes
Principle 5: Encourage Lifestyle Modification for People with Diabetes
Principle 6: Address Overweight and Obesity in the Management of Diabetes
Principle 7: Individualize Blood Glucose Management for People with Diabetes
Principle 8: Provide Multifactorial Cardiovascular Disease Risk Reduction
Principle 9: Detect and Monitor Diabetes Microvascular Complications and Provide Treatment to Slow Their Progression
Principle 10: Consider the Needs of Special Populations with Diabetes

### 5.2 Strengthening the health care systems

The quality crisis of the Indian health care sector is well recognized. The required public health facilities in India count to about 74,150 health centers per million population, but the existing number comes to only half of the original requirement. Many Indian states lack laboratories for testing and more than half of existing laboratories are not properly equipped or staffed. Hence, there is an urgent need to build a well organized infrastructure and make existing healthcare facilities healthier to improve quality of diabetes care and outcomes.

### 5.3 Use of affordable technologies

In India, several care-delivery models for a range of chronic illnesses have been developed to achieve quality care. Some approaches include Cardiovascular Risk Reduction in South Asia (CARRS) diabetes care delivery model for tertiary care facilities, which had been designed as the quality improvement strategy by the All India Institute of Medical Sciences (AIIMS), New Delhi the New Delhi-based Centre for Chronic Disease Control (CCDC) and Public Health Foundation of India (PHFI), and Atlanta-based Emory University. In 2016, Ali et al. compared with usual care in a poorly-controlled diabetes setting (1146 PWD [575 intervention; 571 usual care]) for 28-months and found that 18.2% of intervention participants achieved an HbA1c <7.0% and BP < 130/80mmHg compared to usual care participants [36]. The CARRS model demonstrated the importance of low-cost strategies to achieve diabetes care goals and to improve the quality of life in patients with poorly controlled diabetes.

The AIIMS and CCDC have developed mHealth based digital clinical decision support software (DSS) tools for improving healthcare delivery in various clinical settings. A mobile health-based electronic decision support system, known as mPower Heart mHealth System comprising of DSS integrated Electronic Health Record (EHR), was used primarily by trained nurse care coordinators in the out-patient clinics. mHealth/e-Health based digital clinical DSS model was adopted for state-wide implementation in 56 health facilities throughout the Indian states of Tripura and Mizoram and more than 45,000 individuals with hypertension/diabetes have been treated using DSS.

Diabetes Tele Management System® (DTMS®) originally developed and practiced at Jothydev's Diabetes Research Centre since 1997 has been found to be advantageous in many aspects of diabetes management such as in frequently titrating the doses of medications, drastically reducing the frequency of hospital visits, providing cost-effective treatment, and advices on diet and lifestyle to achieve the customized treatment goals. A study which analyzed the impact of DTMS® in glycemic control had reported that frequent telemedicine follow-ups based on self-monitoring of blood glucose (SMBG) aids in slow and steady titration of drug dose and reducing the risk of serious hypoglycemia [37]. A retrospective study, which evaluated the cost-effectiveness of the telemedicine program, DTMS® in T2DM patients with a six month follow-up data revealed that telemedicine for diabetes management is cost-effective and safe [37]. DTMS® had also found to be effective in preventing microvascular and macrovascular complications in 93.5% of the type 2 diabetes subjects [38]. All these studies emphasize the potential benefits of implementing and practicing affordable technologies such as telemedicine especially in treating the low economic category.

International guidelines recommend diabetes technologies to maintain quality of life and prevent the future cost of treating diabetes. Hence, the government should take necessary steps to avail effective use of diabetes technologies to reduce the diabetes treatment burden.

### 5.4 Public-private partnership

Public-Private partnership is one of the proven strategies to improve access to healthcare, especially in remote areas. Several reasons stress the establishment of private-public partnerships in diabetes care. Firstly, more than 80–90% of the qualified doctors involved in the treatment of diabetes and associated disorders work in the private sector and is one of the reasons why patients prefer private sector, especially affordable patients. Secondly, it is very much essential that a public-private partnership is required for both clinical care and for research as most of the original research publications in diabetes from India come from the private institutions involved in original research. So a public-private partnership for diabetes care and research will have numerous beneficial effects. Some projects such as “Changing Diabetes Barometer and Changing Diabetes in Children” are running successfully in India. The Public Health Foundation of India has tied up with West Bengal and Madhya Pradesh governments to train public sector doctors in diabetes care. These models should be encouraged in resource-constrained areas.

### 5.5 Health care governance

Government initiatives are encouraged to create guidelines on diabetes management, provide funds for public awareness programs, and to ensure an uninterrupted supply of medicines and diagnostic services to PWD. Around the world, various governments have conducted different types of patient education programs and have also achieved positive health outcomes among PWD. In India, similar efforts and services are required at the grass-root level to set quality standards, underpin clinical governance, and to contain the new-age diabetes pandemic. Regular programs should be organized wherein senior doctors from secondary/tertiary level health facilities and medical colleges should mandatorily visit rural areas to help peripheral health workers improve their knowledge and skills.

### 5.6 Universal screening for gestational diabetes mellitus (GDM)

Wide variability in reported prevalence estimates for GDM in India, varying from less than 4% to nearly 18% which refers to the variable screening procedures [39]. The treatment of GDM is suboptimal in India since the patients are not getting treated by the speciality team which is quite essential to prevent the progression to type 2 diabetes. Although the Government of India endorses Diabetes in Pregnancy Study Group India (DIPSI) criteria for GDM diagnosis, real success depends on the commitment level and knowledge of healthcare providers [40, 41].

**Figure 1:**
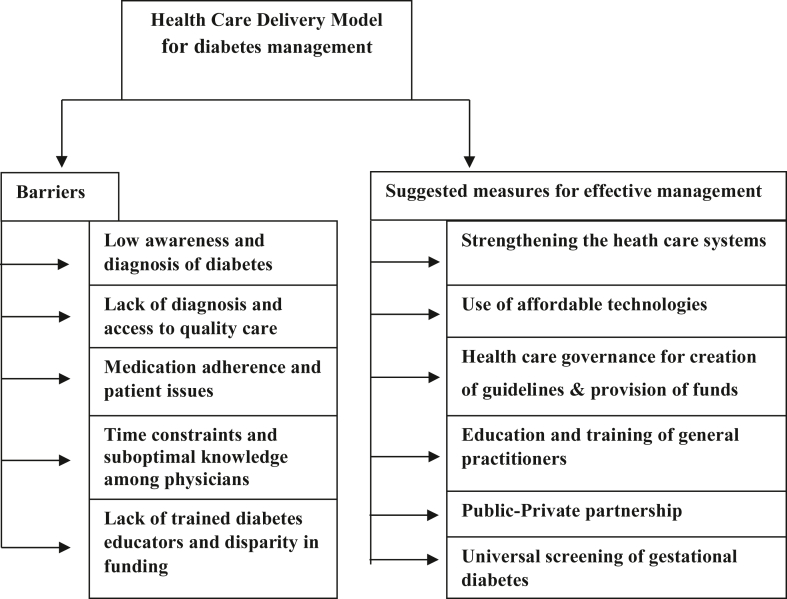
HCD Model for effective diabetes management.

The barriers to the health care delivery model for diabetes management and its suggestive measures are summarized in Figure 1.

## 6 Conclusion

Focus on diabetes education, proactive physician participation, extensive research and a well-planned healthcare system oriented towards diabetes care, form the main areas of desired intervention. Universal screening of diabetes and primary health care possible through public-private partnership may be appropriate in the Indian context. Healthcare professionals should update themselves with recent advances in diabetes diagnosis and treatment and engage in educating patients and the general population. Another welcome initiative on the part of the government will be to subsidize the treatment and care of PWD. Thus a combined effort from patients, healthcare professionals, government, and NGOs can only help to tide over the situation.

## Declarations

### Author contribution statement

All authors listed have significantly contributed to the development and the writing of this article.

### Funding statement

This research did not receive any specific grant from funding agencies in the public, commercial, or not-for-profit sectors.

### Data availability statement

No data was used for the research described in the article.

### Declaration of interests statement

The authors declare no conflict of interest.

### Additional information

No additional information is available for this paper.
